# Appropriateness ratings of everyday behaviors in the United States now and 50 years ago

**DOI:** 10.3389/fpsyg.2023.1237494

**Published:** 2023-10-09

**Authors:** Kimmo Eriksson, Pontus Strimling, Irina Vartanova

**Affiliations:** ^1^School of Education, Culture and Communication, Mälardalen University, Västerås, Sweden; ^2^Institute for Futures Studies, Stockholm, Sweden; ^3^Institute for Analytical Sociology, Linköping University, Norrköping, Sweden; ^4^Department of Women’s and Children’s Health, Uppsala University, Uppsala, Sweden

**Keywords:** social norms, everyday behaviors, externalities, internalities, values, norm shifts

## Abstract

**Introduction:**

A crucial aspect of social norms pertains to determining which behaviors are considered appropriate. Here we consider everyday behaviors. Some everyday behaviors are rated as more appropriate than others, and ratings of the appropriateness of a given behavior may vary over time. The objective of this study is to elucidate the reasons behind variation in appropriateness ratings of everyday behaviors in the United States. Our theory focuses on how the evaluation of the appropriateness of a behavior is influenced by its potential for externalities and internalities, and how this influence may cause a change in norms over time.

**Method:**

Employing a preregistered design, we asked American participants to rate 37 different everyday behaviors based on their appropriateness in a range of common situations, as well as their potential negative externalities (e.g., being loud, being aggressive, taking up space) and positive internalities (e.g., pleasurability). Changes over time were calculated as the difference between mean ratings obtained in this study and ratings of the same behavior in a similar study conducted 50 years ago.

**Results:**

As expected, overall appropriateness ratings of everyday behaviors are associated both with their externalities and their internalities, so that the least appropriate behaviors tend to have considerable potential for negative externalities and little potential for positive internalities. Moreover, behaviors that have considerable potential for negative externalities are perceived as less appropriate now than 50 years ago.

**Discussion:**

By describing how social norms for everyday behaviors depend on the externalities and internalities of behaviors, this study contributes to theories about the emergence and change of social norms.

## Introduction

Even though our everyday lives are governed by social norms, it is far from well understood why certain behaviors are deemed more appropriate than others. Research in social psychology seldom examines variations across behaviors. An exception to this trend is a study conducted by Price and Bouffard (1974), which explored the appropriateness ratings of 15 common behaviors in 15 different situations among a sample of American college students. Notably, this study departed from the usual focus on individual differences and instead examined distinctions between behaviors and situations—an approach also reflected in works by Price (1974), Genereux et al. (1983), Pervin and Furnham (1987), and Gelfand et al. (2011). Price and Bouffard (1974) found that variance in ratings was primarily attributed to differences between behaviors and situations, rather than variations among raters. Aggregating ratings across situations and raters revealed notable discrepancies in overall appropriateness ratings among behaviors. For instance, the overall appropriateness rating for “shouting” was significantly lower than that for “eating.” Consequently, Price and Bouffard (1974) argued that the overall appropriateness of a behavior should be considered an inherent characteristic of the behavior itself.

In this study, we delve into the origins of overall appropriateness. We propose that behaviors are judged as inappropriate if they possess characteristics that are generally valued negatively in that society. Moreover, we contend that a small number of such characteristics can account for differences in appropriateness ratings across numerous behaviors. Additionally, we hypothesize that changes in values over time lead to corresponding shifts in appropriateness ratings. Our reductionist approach suggests that a behavior can be meaningfully represented by measures of a few key properties. The objective of this paper is to explore this approach empirically. We employ a series of four steps: First, we propose a concise set of characteristics of behaviors: their potential for creating negative externalities (i.e., negative consequences for bystanders) and positive internalities (i.e., a positive experience for the actor). Second, we assess the perceived presence of these characteristics across a wide range of everyday behaviors in the United States. Third, we investigate whether these characteristics can account for variations in appropriateness ratings between behaviors in the United States. Finally, we examine whether these characteristics predict how appropriateness ratings of everyday behaviors have changed since the pioneering study of Price and Bouffard (1974).

### Defining everyday behaviors

Following Price and Bouffard (1974), our focus is on everyday behaviors. In the original study, a group of students was asked to keep a detailed diary for a day. From these diaries, the researchers extracted a set of 15 acts (run, talk, kiss, write, eat, sleep, mumble, read, fight, belch, argue, jump, cry, laugh, and shout) that they labeled as everyday behaviors. We suggest a formal definition: An everyday behavior is a conscious act such that at least some people do it regularly, most people could do it if they wanted to, and it can, in principle, be done in almost any location. Note that these criteria exclude one of the original behaviors, sleeping, as people are not conscious when they sleep. Arguably, other rules apply to unconscious people.

### Social norms and appropriateness ratings

The concept of social norms is multi-faceted. It encompasses behavioral regularities, expectations of others’ behavior, sanctions for deviant behavior, and ideas about how one should behave (e.g., Brennan et al., 2013). It is to the latter, injunctive, aspect of social norms that appropriateness ratings speak. Ratings of the appropriateness of various behaviors are often used to compare the strength of norms across different societies (Gelfand et al., 2011; Eriksson et al., 2021a,b). Here we are instead interested in how ratings compare between different behaviors within one society.

### Theories of norm emergence

A key question for theories of social norms is why norms emerge in the first place (Hechter and Opp, 2001). In a brief review of classical thinking in this area, Gelfand et al. (2017, p. 801) distinguish between a perspective where norms are “thought to arise from cultural idiosyncrasies” and an opposing “functionalist” perspective. Of course, it may be that different explanations are required for different norms. For example, certain societies have taboos against certain foods or drinks that are consumed with gusto in other societies; this is arguably a case of cultural idiosyncrasies. On the other hand, a functionalist perspective seems more apt to explain why cooperative behavior is regarded as good in societies across the world (Curry et al., 2019). Indeed, many norms are thought to be cooperation norms at heart, that is, they are thought to emerge to mitigate negative externalities and promote positive outcomes (Hechter and Opp, 2001; Bicchieri, 2005; Roos et al., 2015; Richerson et al., 2016). In line with this “instrumental” perspective on norms, research on social norms frequently focuses on antisocial vs. prosocial behaviors (Malti and Krettenauer, 2013; House, 2018). One of the goals of the present study is to examine to which extent the instrumental perspective can also account for why appropriateness ratings vary across different everyday behaviors.

### The potential for negative externalities

Importantly, everyday behaviors are not prosocial or antisocial in themselves. The context matters. To illustrate, let’s consider the act of shouting. Shouting can be considered prosocial in specific situations, such as when it is necessary to warn others of some threat. In many other situations, shouting is more likely to be perceived as antisocial. Arguably, this is due to a specific characteristic of shouting: it is loud. Loudness infringes on the sonic environment of bystanders. The loudness of shouting has the *potential* to create negative externalities in contexts where there are bystanders who are engaged in other activities or who are seeking a quiet environment.

Averaged across different contexts, a louder behavior will generate greater negative externalities than a quieter behavior, assuming all other factors are equal. Assuming that negative externalities affect people’s perceptions of behaviors, as the instrumental perspective on norms suggests, we may therefore expect behaviors to be regarded as overall less appropriate the louder they are.

Note that loudness is just one example of a behavioral characteristic that can produce externalities. Another example is behaviors that occupy physical space, potentially infringing on the environment of bystanders. A third example is behaviors that exhibit aggression, thereby threatening others and demanding their attention. Instead of trying to capture all negative externalities, we shall focus on these three examples and see how far that will get us. These examples of externalities have two properties that make them especially likely to have observable influence on appropriateness ratings of everyday behaviors.

First, the potential that loudness, occupancy of space, and aggressiveness have to generate negative externalities is fairly *universal*, that is, independent of who the bystanders are. This is because they interact directly with human biology, in contrast to negative externalities caused by violation of cultural sensitivities (e.g., based on religion or nationalism). Whether the latter externalities arise will not only depend on the behavior itself but also on the specific identity of the bystanders, so the effect on appropriateness ratings will be noisy and more difficult to detect. While different cultures may well differ in the *extent* to which they experience, say, loudness as a negative externality, we assume the direction to be universal (i.e., a sufficiently loud and unwelcome noise is experienced negatively by people everywhere).

Second, loudness, occupancy of space, and aggressiveness all show a great deal of *variation across everyday behaviors*. In other words, it is easy to think of several everyday behaviors that are, say, loud, as well as several that aren’t. By contrast, almost no everyday behaviors in the United States create, say, a bad smell. Even though the negative externality of a bad smell may be quite universal, its effect on appropriateness ratings will be difficult to detect if almost no behaviors cause bad smell.

For the same reason, we do not study positive externalities. While studies of cooperation norms often focus on helping behaviors, it seems to us that clear positive externalities are rare for the behaviors we count as everyday behaviors.

### The potential for positive internalities

In addition to externalities, there are reasons to believe that people also consider the *internalities* of behavior, that is, the value the behavior has for the actor. Here we rely on the literature on cooperation in economic games. According to theories of social/moral preferences, people take the balance of the value to others and the value to themselves into account when making decisions (Fehr and Schmidt, 1999; Van Lange, 1999; Bolton and Ockenfels, 2000), and they perceive that balancing the values for others and the self is morally right (Capraro and Perc, 2021). While these theories formally apply to economic games, these games are in turn assumed to represent a much wider scope of situations in which actors have different interests. We therefore expect people to take not only externalities but also internalities into account then they judge how appropriate an everyday behavior is.

In the context of externalities, we argued that everyday behaviors impose costs on bystanders through their sensory systems. Conversely, regarding internalities, certain behaviors directly engage with the agent’s internal reward system (Schultz, 2015). In other words, an act such as eating may inherently possess pleasurable qualities. For this reason, we would expect eating to be rated as more appropriate than behaviors that are less pleasurable but otherwise similar.

### All norms cannot be explained by externalities and internalities

Our proposal is that externalities and internalities are major factors behind norms. Note that this does not exclude the existence of other factors. For example, consider norms about sex. It is typically pleasurable to have sex, and the externalities of having sex do not seem to be worse than for other noisy, physical behaviors. Yet there are very strong norms against having sex in public. Note that if people would have especially loud or unpleasurable sex in public, it seems likely that bystanders would find that especially inappropriate. Thus, it is not the case that externalities and internalities do not apply. Rather, norms against public sex depend on some additional factors too. Our working assumption is that such additional factors have a limited scope. In other words, the proposal we want to test is that a few general kinds of negative externalities and positive internalities are sufficient to account for much of the variation in appropriateness ratings across everyday behaviors.

### Change over time in everyday norms

Our theory assumes that when people judge the appropriateness of behaviors, they take externalities and internalities into account. Judgments likely involve both a direct process, where people judge a behavior directly based on its externalities and internalities, and an indirect social process, in which people learn what is appropriate from other people who in turn, either directly or indirectly, base their judgments on externalities and internalities. Note that the process cannot be entirely indirect; for an effect to arise at all, some judgments must be directly influenced by externalities and internalities.

An important point is that the process may lead to norms changing over time. Here we draw on the moral argument theory (Strimling et al., 2019). According to this theory, individuals may change their judgment of a behavior when exposed to an argument that resonates with them. The theory further assumes that the arguments that most reliably resonate with people, at least in the United States, concern whether the behavior is harmful, and whether it is fair. Thus, when discussing the morality of various behaviors, individuals who currently accept a behavior is more likely to be swayed by an argument of the type “but it is harmful/unfair” than by an argument of the type “but it is against tradition/religion.” Strimling et al. (2019) presented a mathematical model of the dynamic effects of this mechanism, predicting that those judgments that are justified by arguments based on harm and fairness will over time become gradually more common in the population. To test this prediction, the researchers selected a broad set of morality norms (about gender roles, abortion, freedom of speech, etc.) for which the change over the last half-century was known, and examined which arguments these norms were associated with. As predicted, norm strength had increased over time specifically for those morality norms that were supported by arguments concerning harm and fairness.

In sum, the moral argument theory posits that change in morality norms is determined by the kinds of arguments that apply to judging a certain behavior as inappropriate. The same theory could be applied to everyday norms as well. Namely, the kind of universal negative externalities that we study here—generated by loudness, occupancy of space, and aggressiveness—can easily be conceived as harm or unfairness to bystanders. From the moral argument theory, we then obtain the prediction that norms against everyday behaviors in the United States will have become stronger specifically for those behaviors that have a clear potential for negative externalities. In other words, we expect that everyday behaviors with clear negative externalities will receive worse appropriateness ratings today than a half-century ago.

What about positive internalities? This kind of argument was not included in prior studies of the moral argument theory. It is possible that positive internalities work in the same way as negative externalities, so that the possession of positive internalities will contribute to a behavior being rated as increasingly appropriate over time. However, it may also be that positive internalities seldom are voiced as an argument, but mainly serve as an internal judgment heuristic (“if I enjoy doing it, it is an okay thing to do”). In that case, they will not drive a change in norms over time. Thus, the moral argument theory does not yield a clear prediction on whether the positive internalities of everyday behaviors contribute to change in their appropriateness ratings.

### Hypotheses

Above we have outlined the idea that behaviors differ in their potentials to produce externalities and internalities, and that these potentials affect how people ate the appropriateness of a behavior. Our first hypothesis is that much of the variation in the overall appropriateness of everyday behaviors can be reduced to these potentials.

*Hypothesis 1.* Between-behavior differences in overall appropriateness in the United States is in part explained by between-behavior differences in the potential to produce negative externalities (as measured by the degrees to which a behavior is loud, aggressive, and takes up space), which has a negative influence on overall appropriateness, and positive internalities (as measured by the degrees to which a behavior is pleasurable), which has a positive influence on overall appropriateness.

Our second hypothesis is that externalities, and possibly also internalities, determine how appropriateness ratings change over time.

*Hypothesis 2.* Over the last 50 years, overall appropriateness ratings in the United States have decreased especially for those everyday behaviors that possess high potential to produce negative externalities. It is also possible that overall appropriateness ratings have increased for those behaviors that possess high potential to produce positive internalities.

In these hypotheses, it is important to note that we consider the potentials to produce externalities and internalities as inherent properties of the behavior itself. In practical terms, these potentials are measured by aggregating subjective ratings from a sample of raters. Implicitly, we assume that there is a general consensus regarding these potentials. For instance, regardless of the specific sample of raters used, we assume that shouting will consistently be rated as louder than eating, and that the potential for pleasurability will be rated higher for eating compared to shouting. We present this assumption as a hypothesis.

*Hypothesis 3.* Differences between everyday behaviors in the degrees to which they, in the United States, are rated as pleasurable, loud, aggressive, and taking up space, are largely independent of the sample of raters used.

We have similarly assumed that overall appropriateness is a property of the behavior itself, while in practice it is measured through aggregation of ratings not only across a sample of raters but also across a sample of situations. Our assumption requires that the results are largely independent of both kinds of samples. In their discussion of overall appropriateness as a behavioral dimension, Price and Bouffard (1974) implicitly assumed such independence. However, they did not explicitly test it in their data, nor did they provide an argument for why sample independence would arise. Our theory, together with Hypothesis 3, provides such an argument. We therefore state sample independence of overall appropriateness as a final hypothesis.

*Hypothesis 4.* Differences between everyday behaviors in the degrees to which they, in the United States, are rated as overall appropriate is largely independent of (a) the sample of raters and (b) the sample of situations.

## Materials and methods

This is a preregistered study. The hypotheses, the data collection, and the primary analyses were preregistered at AsPredicted (Hypotheses 1 and 2^1^; Hypotheses 3 and 4^2^). We report all measures, and exclusions in this study. We used R version 4.2.2 (R Core Team, 2022) for data processing, analysis, and visualization including packages dplyr (Wickham et al., 2023a), tidyr (Wickham et al., 2023b), ggplot2 (version 3.4.0, Wickham, 2016), and ggrepel (Slowikowski, 2022). All data, the codebook, the analysis code, and the questionnaire are available at OSF.^3^

Data was collected using an online questionnaire. The purpose of the study was clearly described to participants and informed consent was obtained. Participants were completely anonymous, and the study did not seek to influence them in any way. Studies fulfilling these criteria are exempt from ethics review according to regulations in Sweden (the country from which the study is conducted).

### Selection of behaviors

Our hypotheses concern systematic variation across behaviors. Thus, the study needs to include a sufficiently large set of behaviors. We expected a large effect size: a correlation above 0.50 between overall appropriateness and the net possession of potential for externalities-internalities. To achieve 80% power for detecting this effect size at a significance threshold α = 0.05, a sample of at least 29 behaviors is required. The study of Price and Bouffard (1974) used the aforementioned set of 15 behaviors of which we used all but sleeping. From a related study by Gelfand et al. (2011), we obtained another five behaviors: sing, curse, flirt, blow one’s nose, listen to music on headphones. To obtain additional everyday behaviors we organized a brainstorming session with two research assistants, which resulted in a list of suggestions. From this list we excluded behaviors that did not fully satisfy our definition, as well as some behaviors that we judged to be too ambiguous or too similar to another behavior. To make sure that appropriateness ratings capture the relevant differences in social judgments across behaviors, we also did not include behaviors that are clearly socially obliged (not just appropriate). After adding the remaining behaviors to those obtained from prior studies, we arrived at a total list of 37 everyday behaviors that we use in this study. See Table 1.

**TABLE 1:** Average ratings of 37 different everyday behaviors (sorted by increasing overall appropriateness).

Behavior	Overall appropriateness	Is loud	Is aggressive	Takes up space	Pleasurable
Fight	0.71	4.55	4.82	4.05	1.39
Spit	1.36	2.18	3.86	2.23	1.68
Argue	2.21	4.46	4.52	2.50	1.46
Run	2.45	2.86	2.97	3.88	3.29
Fart	2.47	3.57	2.65	2.04	2.52
Shout	2.83	4.77	4.32	2.03	2.11
Do a jigsaw puzzle	3.00	1.34	1.35	3.99	3.85
Curse	3.08	3.91	4.28	1.87	2.48
Sit on the floor/ground	3.20	1.30	1.74	3.92	2.95
Jump	3.24	2.94	2.67	3.54	3.13
Belch	3.25	4.09	2.78	2.06	2.57
Pray audibly	3.40	3.82	2.31	2.11	2.88
Play cards	3.53	2.33	1.63	3.69	4.06
Bring a dog	3.75	3.31	2.07	4.15	3.95
Whistle	3.77	4.29	2.32	1.64	3.20
Work on a laptop	3.81	2.00	1.48	3.61	3.03
Cry	4.05	3.70	1.99	1.79	1.84
Talk on the phone	4.08	4.10	2.25	2.17	3.41
Dance	4.10	3.10	2.20	4.06	4.22
Sing	4.11	4.34	1.92	2.03	4.05
Flirt	4.37	2.67	2.44	2.05	4.04
Kiss	4.43	2.13	2.17	2.48	4.52
Mumble	4.58	2.09	1.70	1.51	2.39
Blow one’s nose	4.58	4.02	2.25	2.04	2.38
Write	4.91	1.38	1.37	2.38	3.86
Listen to music on headphones	5.01	2.07	1.45	1.60	4.52
Fiddle with one’s phone	5.12	1.75	1.54	1.81	3.71
Read	5.28	1.32	1.15	1.92	4.32
Take a selfie	5.44	1.82	1.79	2.90	3.56
Sigh	5.63	2.66	2.11	1.47	2.79
Chew gum	5.88	2.73	1.67	1.30	3.67
Eat	5.95	2.70	1.52	2.91	4.55
Hold hands	6.28	1.32	1.42	2.26	4.42
Laugh	6.82	4.25	1.88	1.61	4.66
Talk	7.06	3.85	2.12	2.09	4.07
Wave to a friend	7.11	1.38	1.41	1.74	4.30
Drink water	7.70	1.66	1.23	1.69	4.19

### Selection of situations

We use the same 15 situations as Price and Bouffard (1974): in class, on a date, on a bus, at a family dinner, in the park, in church, at a job interview, on a downtown sidewalk, at the movies, in a bar, in an elevator, in a restroom, in one’s own room, in a dormitory lounge, at a football game.

### Participants

Participants in the United States were recruited online using Prolific during the period January 17–20, 2023. The recruitment goal was a sample of 400 participants, reasonably balanced with respect to gender (women vs. men), age (above vs. below 40 years), ideological affiliation (liberals vs. conservatives), and education (college educated vs. not). The final sample consisted of 555^4^ participants (50% women and 2% of other gender, 38% above 40 years, 56% liberals, 54% college educated).

### Justification of sample size

In a similar study on arguments for moral opinions, Vartanova et al. (2021) collected a total of 100 ratings per item, which was sufficient to capture the variation in ratings across items and to establish its similarity between subsamples of participants (e.g., women vs. men). We therefore set a similar goal of 100 ratings of each characteristic per behavior. To avoid fatigue, each participant only rated a random selection of 10 behaviors (out of 37). Thus, a sample size of 400 participants would be sufficient to reach the goal of at least 100 ratings of each characteristic per behavior.

### The questionnaire

After obtaining informed consent, we asked participants for their age and country of residence, and whether they committed to thoughtfully providing their best answers to the questions. Participants were deemed not eligible for the study if they reported an age below 18 years or reported a country of residence other than the United States or did not commit to providing their best answers.

In the first main part of the questionnaire, participants were asked “For each behavior below, please indicate how appropriate it would be < in class/on a date/on a bus/etc. >.” They rated the behaviors five times for different situations, which were selected at random for each participant from the full set of situations. Following Price and Bouffard (1974), ratings were given on a ten-point scale from 0 to 9, anchored at 0 = The behavior is extremely inappropriate in this situation and 9 = The behavior is extremely appropriate in this situation.

In the second part of the questionnaire, participants were asked “For each of the behaviors below, to what extent do you agree that it < takes up space/is loud/is aggressive/is pleasurable > .” They rated the behaviors four times, once for every characteristic. Ratings were given on a five-point Likert scale (strongly disagree, somewhat disagree, neither agree nor disagree, somewhat agree, strongly agree), coded from 1 to 5.

The questionnaire ended with questions about level of education, gender, and political views on a seven-step scale from extremely liberal to extremely conservative.

### Analysis

The following analyses were preregistered.

*Hypothesis 1*

For each behavior, we estimate its current overall-appropriateness rating in the United States by the average of all ratings of the behavior across all 15 situations. We similarly estimate the behavior’s average possession rating for each characteristic.

Using the 37 behaviors as the units of analysis, we first calculate the raw correlations between overall-appropriateness ratings and possession ratings. This is done to ascertain that all signs are as expected: negative correlations with the negative externalities “loud,” “aggressive,” and “takes up space,” and a positive correlation with the positive internality “pleasurable.”

We reverse-code the possession ratings for negative externalities so that higher scores refer to less negative externalities. We then calculate two indices for each behavior: an *externalities index* calculated as the average of the three externalities ratings, and an *externalities-internalities index* calculated as the average of all four possession ratings. We test Hypothesis 1 in two steps. In the first step, we perform a simple linear regression of overall appropriateness on the externalities index-internalities, expecting a positive effect. In a second step, we examine the independent effects of externalities and internalities by performing a linear regression of overall appropriateness on two regressors: the internalities rating and the externalities index. We accept the hypothesis if the effects are significant at *p* < 0.05.

*Hypothesis 2*

The overall-appropriateness levels in the United States 50 years ago are obtained from the study of Price and Bouffard (1974). Data from both points in time are therefore only available for 14 behaviors. This gives us very limited power to confirm this hypothesis, but it is the best we can do. We test the hypothesis in two steps, similar to Hypothesis 1, but with the difference that we are here analyzing variation in the *change* in appropriateness ratings.

In the first step, we analyze whether appropriateness ratings change more in the negative direction the worse the externalities-internalities index of the behavior is. Thus, change scores (current ratings minus ratings from 50 years ago) should exhibit a positive slope with respect to the externalities-internalities index. To test the hypothesis, we therefore perform a linear regression of change scores on the externalities-internalities index across the 14 behaviors (This is equivalent to estimating the interaction between time and externalities-internalities in a linear model that takes into account that appropriateness ratings refer to the same set of behaviors at both time points).

In the second step, we instead regress the change scores on two regressors, the internalities rating and the externalities index, to estimate their independent effects on how appropriateness ratings change over time (This analysis was not preregistered).

*Hypothesis 3*

We test this hypothesis using four different splits of the full sample of 400 raters into two non-overlapping subsamples, “A” and “B”: male vs. female^5^, above vs. below 40 years^6^, with vs. without college degree, and liberal vs. conservative ideological affiliation. For every split we aggregate characteristic-possession ratings per behavior in each subsample. For a given characteristic, we accept the hypothesis if, in every split, we find a Pearson correlation between the “A” ratings and the “B” ratings of at least 0.71 (corresponding to at least 50% of the variance of aggregated ratings in one subsample accounted for by the aggregated ratings in the other subsample).

*Hypothesis 4*

We test Hypothesis 4a by the same method as Hypothesis 3, substituting overall-appropriateness for characteristic-possession and aggregating over the subsample of raters as well as over the full sample of situations. We accept the hypothesis if, in every split, we find a Pearson correlation between the “A” ratings and the “B” ratings of at least 0.71.

To test Hypothesis 4b in a corresponding way, we need to repeatedly split the full sample of situations into non-overlapping subsamples. As there are no corresponding demographic variables for situations, we make four random splits of the 15 situations into an “A” sample of size 7 and a “B” sample of size 8. We aggregate appropriateness ratings over the subsample of situations as well as over the full sample of raters. and use them to calculate two separate overall-appropriateness ratings of each behavior by aggregating their ratings across all situations in the subsample and across the entire sample of raters. We accept the hypothesis if, in every split, we find a Pearson correlation between the “A” ratings and the “B” ratings of at least 0.71.

## Results

### Test of Hypothesis 1

**FIGURE 1:**
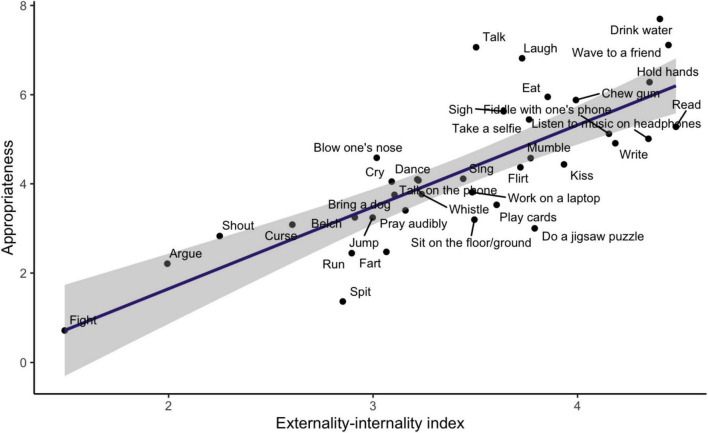
The overall appropriateness ratings of 37 everyday behaviors in the United States, plotted against their externality-internality index.

The overall appropriateness and aggregated ratings of potentials for externalities and internalities of the 37 everyday behaviors are presented in Table 1. As expected, the overall appropriateness of everyday behaviors is negatively correlated with their loudness, *r* = −0.36, aggressiveness, *r* = −0.71, and taking up of space, *r* = −0.47, and positively correlated with their pleasurability, *r* = 0.70. A linear regression of overall appropriateness on the externality-internality index yields a significant positive effect, *B* = 1.83 95% CI [1.34, 2.33], β = 0.79 *t* = 7.52, *p* < 0.001, explaining 62 percent of the variance in overall-appropriateness ratings across behaviors (see Figure 1). When the externalities index and the internalities score are entered as separate regressors, significant independent effects are observed of both externalities, *B* = 1.09 95% CI [0.49, 1.70], β = 0.47, *t* = 3.68, *p* < 0.001, and internalities, *B* = 0.71 95% CI [0.27, 1.15], β = 0.42, *t* = 3.26, *p* < 0.001, together explaining 63 percent of the variance. We conclude that both externalities and internalities contribute to the overall appropriateness of a behavior. In sum, Hypothesis 1 was supported.

### Test of Hypothesis 2

**FIGURE 2:**
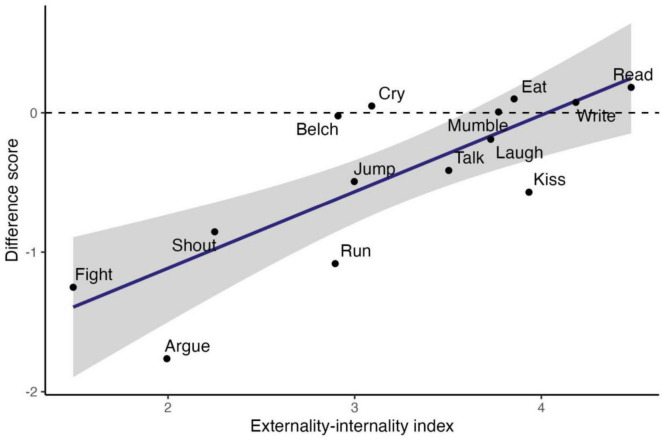
Change scores (2,023 levels minus 1,974 levels) for overall appropriateness ratings of 14 everyday behaviors in the United States, plotted against their externality-internality index. Note that changes are typically in the negative direction (i.e., below the reference line at zero).

As expected, behaviors were generally rated somewhat less appropriate in 2023 than in 1974, with the greatest decreases in overall appropriateness observed for the behaviors with the lowest scores on the externality-internality index (fighting and arguing). See Figure 2. A linear regression of the difference scores for overall appropriateness on the externality-internality index yielded a significant positive effect, *B* = 0.55 95% CI [0.29, 0.81], β = 0.80, *t* = 4.58, *p* < 0.001, explaining 64% percent of the variance in difference scores across behaviors. When the externalities index and the internalities score are entered as separate regressors of change scores, there is a significant independent effect of externalities, *B* = 0.58 95% CI [0.25, 0.92], β = 0.84, *t* = 3.83, *p* < 0.001, but no significant effect of internalities, *B* = −0.01 95% CI [−0.25, 0.23], β = −0.02, *t* = −0.08, *p* = 0.94. Thus, the findings support the hypothesis that negative externalities drive norm change, but do not support that norm change is influenced by positive internalities.

### Tests of Hypotheses 3 and 4

When the full sample of 555 participants was split into two non-overlapping subsamples, based on either gender, age, education, or ideology, the Pearson correlation between ratings aggregated in each subsample ranged between 0.97 and 0.99 for loudness, between 0.97 and 0.98 for aggressiveness, between 0.96 and 0.98 for taking up space, between 0.96 and 0.99 for pleasurability, and between 0.97 and 0.99 for overall appropriateness. See Table 2. We conclude that rating differences between behaviors are indeed largely independent of the sample of raters.

**TABLE 2:** Pearson correlations (across 37 behaviors) between aggregated ratings from different subsamples.

Behavioral characteristic	*r*(Male, female)	*r*(Above 40, below 40)	*r*(With, without college degree)	*r*(Liberal, conservative)
Overall appropriateness	0.98	0.98	0.99	0.97
Loudness	0.99	0.97	0.99	0.99
Aggressiveness	0.97	0.98	0.98	0.98
Takes up space	0.97	0.96	0.98	0.96
Pleasurability	0.96	0.97	0.99	0.97

*r*(X, Y) refers to the Pearson correlation between aggregated ratings from the X subsample and aggregated ratings from the Y subsample.

Similarly, when the full sample of 15 situations was split at random into two non-overlapping subsamples, the Pearson correlation between the overall-appropriateness ratings obtained in each subsample ranged between 0.83 and 0.92 across four random splits, well above the 0.71 threshold (We then extended the analysis to 100 random splits, none of which yielded a correlation below the 0.71 threshold). We conclude that sample independence of overall-appropriateness ratings of behaviors also applies to the sample of situations. In sum, Hypotheses 3 and 4 were supported.

## Discussion

This study focused on ratings of the appropriateness of everyday behaviors in the United States. We defined everyday behaviors as conscious acts that exhibit two forms of universality: first, that at least some individuals regularly engage in these acts (and most people could if they desired), and second, that these acts can be performed in nearly any setting in the given society. While the situational context can influence how others perceive the appropriateness of an act, the impact on appropriateness ratings tends to remain relatively constant across different behaviors (Price and Bouffard, 1974). Therefore, averaging ratings of a behavior across various situations allows us to obtain an overall measure of appropriateness. This approach enables us to move beyond narrow considerations of why a specific everyday behavior is appropriate or inappropriate in a given situation and instead focus on determining the factors that determine overall appropriateness levels across different behaviors.

Our hypothesis posited that a behavior’s potentials for externalities and internalities are significant determinants of its overall appropriateness. Supporting this hypothesis, we found that measures of these potentials accounted for a significant portion of the variation in overall appropriateness ratings across a range of everyday behaviors. These findings align with instrumental theories of social norms, which suggest that norms emerge to mitigate negative externalities and promote positive ones (Hechter and Opp, 2001). However, our study goes beyond instrumental theories by demonstrating that social judgments also take internalities into account. This aligns with studies on moral preferences (Capraro and Perc, 2021) and makes sense from a group-level optimization perspective, as the total payoff to the group encompasses both internalities and externalities.

While our reductionist approach to explaining the appropriateness of everyday behavior by focusing on a few key characteristics, such as the potentials for internalities and externalities, proved to be valuable, it is essential to acknowledge that these characteristics do not encompass all aspects relevant to appropriateness. Behaviors may differ in their perceived privacy, religious significance, association with certain individuals, and more. These additional characteristics could be measured and studied for their effects on appropriateness ratings. However, considering that internalities and externalities already accounted for a substantial portion of the variation in appropriateness ratings, the impact of these additional characteristics may be limited.

Our approach in this study is quite original. Unlike studies that view social norms as dichotomous concepts and examine specific cases of norm emergence (e.g., Hechter and Opp, 2001), we examined continuous variation in appropriateness ratings across multiple behaviors. This approach allows for statistical testing of hypotheses regarding differences between behaviors. We believe this is a crucial complement to studies that manipulate social judgments through framing or the provision of information about behavior frequency (e.g., Cubitt et al., 2011; Eriksson et al., 2015; Banerjee, 2016; Lindström et al., 2018). Even in such studies, the effects of social information on social judgments are often overshadowed by intrinsic differences between behaviors. For example, in the study by Lindström et al. (2018), contributing to a public good was consistently rated higher than free-riding, regardless of manipulations of free-riding frequency. Therefore, understanding social norms necessitates consideration of the intrinsic differences between behaviors.

Thanks to the availability of data on how everyday behaviors were rated in the United States in 1974, from the original study by Price and Bouffard (1974), we were able to examine how appropriateness ratings have changed over time. We found a drop in appropriateness ratings related to the behaviors’ potential for negative externalities. Behaviors that have a clear potential for negative externalities, such as fighting and arguing, are now rated as considerably less appropriate than 50 years ago. This finding is consistent with the moral argument theory of opinion change, which describes how norms may change gradually as individuals sometimes change their judgments of behaviors due to exposure to arguments that point out externalities in the form of harm and unfairness to others. As described by Strimling et al. (2019), the aggregated effect of individuals changing their judgments in one direction more often than in the other direction is that population-level exhibit directional change.

In contrast to these dynamic effects of negative externalities, we did not find any evidence of dynamic effects of positive internalities. Our interpretation is that the influence of internalities and externalities goes through different pathways. The dynamic effects of externalities arise because people explicitly use externalities as arguments for judgments of everyday behaviors (“don’t do that, it’s disturbing other people”). Internalities may instead influence ratings mainly as a judgment heuristic (“I like doing that so it is an okay behavior”) and seldom be voiced as an argument.

It is important to note some limitations in this comparison of ratings over time. Firstly, the data from the 1970s were only available for 14 behaviors. As behaviors are the units of analysis, this means that the analysis of the role of potentials for internalities and externalities in rating changes over time is based on just 14 data points. Additionally, the data from the 1970s were obtained from students, whereas the new data came from a more diverse sample of the population. Nevertheless, this difference in sample characteristics is unlikely to drive our findings. In the current study, we found that differences between behaviors in their aggregated ratings were remarkably consistent across various demographic groups in the United States. The results regarding differences between behaviors were virtually identical whether the ratings came from young or old participants, men or women, individuals with or without a college degree, or liberal or conservative participants. This consistency across samples is advantageous for research on this topic and supports the conceptualization of the rated characteristics as inherent to the behaviors; however, note that this study does not answer to what extent ratings of these characteristics are consistent across cultures.

The ability to explain historical changes in appropriateness judgments raises the prospect of predicting how they will evolve in the future. Assuming that the process that drove the observed changes is still ongoing, we predict further decreases in the perceived appropriateness of behaviors with a high potential for negative externalities.

One important limitation of our study is that it exclusively focuses on society-level norms in the United States. It is widely recognized that everyday norms vary in strength across societies (Gelfand et al., 2011), and they may also vary between groups within a society. While not examined in this study, our theory suggests that this variation may stem from underlying differences in the valuation of internalities and externalities. Exploring this variation across cultures and societies is a valuable area for future research.

In conclusion, this study contributes to our understanding of the emergence of social norms by explaining differences in the perceived appropriateness of everyday behaviors. Furthermore, it provides a theoretical and empirical foundation for future investigations into the reasons behind changes in social norms over time and their variations across different cultures.

## Data availability statement

The datasets presented in this study can be found in online repositories. The names of the repository/repositories and accession number(s) can be found below: All data, the codebook, the analysis code, and the questionnaire are available at OSF (https://osf.io/nzgpt/).

## Ethics statement

Ethical approval was not required for the studies involving humans because data was collected using an online questionnaire. The purpose of the study was clearly described to participants and informed consent was obtained. Participants were completely anonymous, and the study did not seek to influence them in any way. Studies fulfilling these criteria are exempt from ethics review according to regulations in Sweden (the country from which the study is conducted). The studies were conducted in accordance with the local legislation and institutional requirements. The participants provided their written informed consent to participate in this study.

## Author contributions

KE and PS conceived the study. KE, PS, and IV designed the study. KE designed the analysis plan and drafted the manuscript. IV implemented the statistical analyses. IV and PS provided critical revisions. All authors gave final approval for publication and agreed to be held accountable for the work performed therein.

## Footnotes

1
https://aspredicted.org/9G7_TK4

2
https://aspredicted.org/P2V_L4Y

3
https://osf.io/nzgpt/

4 By mistake, the main survey did not include “fight” but instead included “pray audibly” twice. We therefore ran an additional data collection in which 150 participants rated “fight” along with four randomly selected behaviors. We also excluded ratings from the second evaluation of “pray audibly” from 30 participants who evaluated the behavior twice.

5 For this analysis we excluded 11 participants who self-identified with other gender.

6 For this analysis we excluded one participant who reported an age of 222.
